# An Updated View of Translocator Protein (TSPO)

**DOI:** 10.3390/ijms18122640

**Published:** 2017-12-06

**Authors:** Nunzio Denora, Giovanni Natile

**Affiliations:** 1 Department of Pharmacy-Drug Sciences, University of Bari “Aldo Moro”, 70125 Bari, Italy; 2 Department of Chemistry, University of Bari “Aldo Moro”, 70125 Bari, Italy

**Keywords:** translocator protein (TSPO), neuroinflammation, steroidogenesis, subcellular targeting

## Abstract

Decades of study on the role of mitochondria in living cells have evidenced the importance of the 18 kDa mitochondrial translocator protein (TSPO), first discovered in the 1977 as an alternative binding site for the benzodiazepine diazepam in the kidneys. This protein participates in a variety of cellular functions, including cholesterol transport, steroid hormone synthesis, mitochondrial respiration, permeability transition pore opening, apoptosis, and cell proliferation. Thus, TSPO has become an extremely attractive subcellular target for the early detection of disease states that involve the overexpression of this protein and the selective mitochondrial drug delivery. This special issue was programmed with the aim of summarizing the latest findings about the role of TSPO in eukaryotic cells and as a potential subcellular target of diagnostics or therapeutics. A total of 9 papers have been accepted for publication in this issue, in particular, 2 reviews and 7 primary data manuscripts, overall describing the main advances in this field.

## 1. Introduction

In eukaryotic cells mitochondria play a vital role as they are involved in the control of oxidative phosphorylation and ATP synthesis and in the regulation of apoptosis and translocation of pro-apoptotic proteins from the mitochondrial intermembrane space to the cytosol [1]. Hence, it is evident that mitochondria can regulate cell survival and that mitochondrial dysfunctions are involved in the onset of several human pathological conditions. For this reason, mitochondria can be considered a potential target for the delivery of therapeutics, although its intracellular localization makes it difficult to reach. Decades of study on the role of mitochondria in living cells have evidenced, in particular, the importance of the 18 kDa mitochondrial translocator protein (TSPO), first discovered in the 1977 as an alternative binding site for the benzodiazepine diazepam in the kidneys. This protein participates in a variety of cellular functions, including cholesterol transport, steroid hormone synthesis, mitochondrial respiration, permeability transition pore opening, apoptosis, and cell proliferation [2,3,4,5,6,7]. In accordance with TSPO’s diverse functions, changes in TSPO expression have been linked to multiple diseases, from cancer to endocrine and neurological diseases. Thus, TSPO has become an extremely attractive subcellular target for (1) early detection of disease states that involve the overexpression of this protein [8,9,10] and (2) selective mitochondrial drug delivery [11,12,13,14]. To date, several studies have been carried out on the synthesis of new structurally diverse TSPO ligands and on the preparation of nanosystems or metal complexes that can be directed to TSPO for diagnosis or therapy [15,16], thus highlighting the great interest of the scientific community in understanding the functions of this translocator protein in both normal and pathological conditions.

Investigation of the functions of this protein, both in vitro and in vivo, has been mainly carried out using high-affinity ligands, such as isoquinoline carboxamides (e.g., PK 11195) and benzodiazepines (e.g., Ro5-4864). For instance, PK 11195 and Ro5-4864 have been used to explore TSPO distribution and function in various tissues and pathologies, thus allowing the mapping of the “peripheral binding site” in almost every tissue examined [17].

As a result of the great interest in the role of TSPO and its potential use as subcellular target, this special issue, entitled, “Translocator Protein (TSPO)”, was programmed to consolidate new knowledge focused specifically on this receptor. A total of 9 papers were accepted for publication in this issue: 2 reviews and 7 primary data manuscripts, focusing on (1) new functions attributable to TSPO; (2) new potent and selective TSPO ligands; (3) the use of ligands as imaging tools for the early diagnosis of diseases characterized by a high expression of TSPO, such as neuroinflammation and TSPO-rich cancers; (4) TSPO targeted nanocarriers that deliver therapeutics and diagnostics; (5) TSPO ligands that could be used to prepare coordination complexes of metallodrugs for use in diagnosis and therapy; (6) TSPO ligands as pro-apoptotic agents that are potentially useful for the treatment of cancers, and, finally; (7) in vitro and in vivo investigations of the ability of TSPO ligands to affect steroidogenesis.

## 2. Articles in This Special Issue

Mitochondria are involved in several metabolic processes, comprising the energy transduction mechanism which requires the transport of specific metabolites across the inner membrane which is achieved through mitochondrial carriers (MCs), a family of nuclear-encoded proteins sharing several structural features [18]. The MCs’ function is to facilitate the exchange of metabolites through the inner mitochondrial membrane (IMM). In this regard, Damiano et al. contributed to this special issue with a review concerning two mitochondrial carriers, such as citrate and carnitine/acylcarnitine transporters, involved in fatty acid metabolism [19]. Interesting new research on the mechanisms involved in the regulation of lipid metabolism in the cell could be sparked from this study.

The study of Gavish and coworkers, also reported in this issue [20], provides a deeper understanding of the overall biological function of TSPO. By applying the microarray analysis of the gene expression in U118MG glioblastoma cells, they discovered that the classical TSPO ligand PK 11,195 can modulate gene expression in this tumor cell line and induce cell morphological changes. In particular, at exposure times of 15, 30, 45, and 60 min, as well as 3 and 24 h, to PK 11,195, changes in gene expression might be associated with several cellular functions including viability, proliferation, differentiation, adhesion, migration, tumorigenesis, and angiogenesis. This was supported microscopically by cell migration, cell accumulation, adhesion, and neuronal differentiation. The authors propose that the modulation in gene expression occurs via mitochondria-to-nucleus signaling; thus, TSPO modulates not only local mitochondrial functions but also nuclear gene expression. Furthermore, Gavish and coworkers identified a novel TSPO ligand, the 2-Cl-MGV-1, able to modulate gene expression of immediate early genes and transcription factors [20]. The results reported in this work highlight the possible effects on cellular and organismal functions induced by TSPO ligands possibly via modulation of nuclear genes expression promoted by mitochondrial TSPO. This type of modulation can influence several vital cell functions, with major implications on the whole organism in health and disease states.

The article of Culty and coworkers in this issue gives further insight into the role of TSPO in living cells [21]. They showed that TSPO is downregulated during gonocyte differentiation, which is indicative of a possible repressive role. Moreover, expression studies in human normal testes confirmed that TSPO is expressed in subsets of adult germ cells, suggesting a function in acrosome formation, while the analysis of tumor samples revealed the upregulation of its mRNA and protein localization in seminoma cells. The authors’ prospect is to investigate the exact role of TSPO in normal spermatogenic cell development (from gonocyte to more mature germ cells) and in testicular cancer.

The investigation of the TSPO functions takes advantage of the use of synthetic TSPO ligands. Veenman and coworkers contributed to this issue with a review on the role of Tetrapyrroles as endogenous ligands for TSPO in comparison with synthetic ligands [22]. Interactions between the 18 kDa translocator protein (TSPO) and tetrapyrroles, including the tetrapyrrole protoporphyrin IX (PPIX), have been studied for several decades in various species. Thus, Veenman et al. give an overview and the future perspectives for research regarding interactions between TSPO and tetrapyrroles. TSPO can be considered a receptor for PPIX, a transporter for tetrapyrroles, and a participant in the regulation of tetrapyrrole metabolism; vice versa, tetrapyrroles can modulate TSPO functions. A better understanding of the structure–function interactions between TSPO and its endogenous ligands such as tetrapyrroles, including PPIX, may aid in the development of new synthetic TSPO ligands as versatile drugs for the treatment of various diseases. In the review, apart from interactions between TSPO and tetrapyrroles, the effects of synthetic TSPO ligands in the context of TSPO–tetrapyrrole interactions are also presented.

Martini and coworkers [23] contributed here with 13 new high affinity TSPO ligands belonging to their previously described *N*,*N*-dialkyl-2-phenylindol-3-ylglyoxylamide (PIGA) class. The new ligands were evaluated for their potential ability to affect the cellular Oxidative Metabolism Activity/Proliferation index, which is used as a measure of astrocyte well-being. The relevance of neurosteroidogenesis in astrocyte well-being was investigated in a human astrocyte model and the positive effect of TSPO-stimulated neurosteroid release on astrocytes well-being was demonstrated. The development of molecules able to stimulate steroid release could represent a therapeutic strategy for central nervous system diseases characterized by astrocyte loss. Furthermore, these ligands may be exploited as pharmacological tools for investigating the autocrine/paracrine roles of neurosteroids in the control of astrocyte metabolism.

In order to develop highly selective and active TSPO ligands for cancer therapy and imaging, Lee and coworkers synthesized a new imidazopyridine-based TSPO ligand (CB256) for coordination to ^99m^Tc and Re [24]. The ^99m^Tc-labeled imidazopyridine-based bifunctional chelate ligand was prepared in one step with good radiochemical yield. The resulting complex showed high stability in vitro. The coordination to tricarbonyl rhenium did not alter the TSPO affinity of CB256. In vitro studies on TSPO-rich tumor cells suggested that the radiolabeled complex may have a potential as SPECT radiotracer for the evaluation of TSPO-overexpressing tissues, thus calling for further in vivo biological evaluation.

In this special issue Laquintana and coworkers present two TSPO ligand-methotrexate conjugates that are potentially useful for the treatment of TSPO-rich cancers, including brain tumors [25]. Methotrexate (MTX) is the drug of choice for the treatment of several cancers, but its permeability through the blood–brain barrier (BBB) is poor, making it unsuitable for the treatment of brain tumors. In contrast, the TSPO ligand-MTX conjugates prepared by these authors showed a high binding affinity and selectivity for TSPO, and a more marked toxicity toward glioma cells than MTX alone. These results confirm the ability of the selected TSPO ligand to transport a hydrophilic drug through the biological membranes and determine its accumulation in target cells overexpressing TSPO. The study of Laquintana and coworkers also demonstrates the effectiveness of the bio-conjugate strategy for bringing two agents with a distinct mechanism of action to cancer cells.

The use of TSPO ligands for preparing coordination complexes of metallodrugs with diagnostic and/or therapeutic potential has also been exploited by Margiotta and coworkers [26], who present here the first Pt(IV) derivative of oxaliplatin carrying a ligand for TSPO. This new Pt(IV) complex has been fully characterized from a chemical point of view and has been tested in vitro against human MCF7 breast carcinoma, U87 glioblastoma, and LoVo colon adenocarcinoma cell lines. The affinity for TSPO receptor, the cellular uptake, and the effect on cell cycle progression were also evaluated. The results obtained by these authors render this new coordination complex very promising in the context of a receptor-mediated drug targeting strategy toward TSPO-overexpressing tumors, in particular the colorectal cancer.

Finally, Papadopoulos and coworkers contributed to this issue with an article concerning the ability of the peptide VLNYYVW, designed on the TSPO’s CRAC (cholesterol recognition/interaction amino acid consensus) domain, to prevent the opening of the mPTP and the release of apoptotic factors in rat brain mithocondria [27]. In addition, the authors showed that the TSPO specific drug ligand PK 11,195 modulates the effects of the CRAC peptide on the induction of mPTP opening and the release of apoptotic factors. These results suggest that TSPO via its C-terminal CRAC domain participates in mPTP function/regulation and apoptosis initiation and that TSPO drug ligands are regulators of this process.

## 3. Conclusions

The high number of papers submitted and ultimately accepted for publication in this special issue attests to the considerable amount of research being conducted on TSPO and TSPO’s role in living cells. TSPO has become an extremely attractive subcellular target for the early detection of disease states (that involve the overexpression of this protein) and for the selective delivery to mitochondria of drugs for diagnostic and therapeutic purposes. Moreover, the effort in the design and synthesis of new, more specific and effective TSPO ligands has been valuable and cannot be neglected.
